# P-1195. Incidence and Diagnosis of Neonatal Herpes Simplex Virus (HSV) Following Transition to In-House Serum Testing

**DOI:** 10.1093/ofid/ofae631.1379

**Published:** 2025-01-29

**Authors:** Elizabeth Patt, Julie A Ribes, Joel I Howard, Katie B Olney

**Affiliations:** University of Kentucky Healthcare, Lexington, Kentucky; University of Kentucky, Lexington, KY; University of Kentucky HealthCare, Lexington, Kentucky; University of Kentucky HealthCare, Lexington, Kentucky

## Abstract

**Background:**

Neonatal Herpes simplex virus (HSV) infection is associated with significant morbidity and mortality, and previously estimated to account for 0.2-0.9% of hospitalizations in infants admitted with sepsis nationally. Recently, our institution transitioned the performance of serum HSV PCR testing in-house to expedite evaluation in neonates admitted with concerns for sepsis. This study was conducted to internally review the impact of in-house testing on neonatal HSV diagnosis and incidence.

**Table 1:**
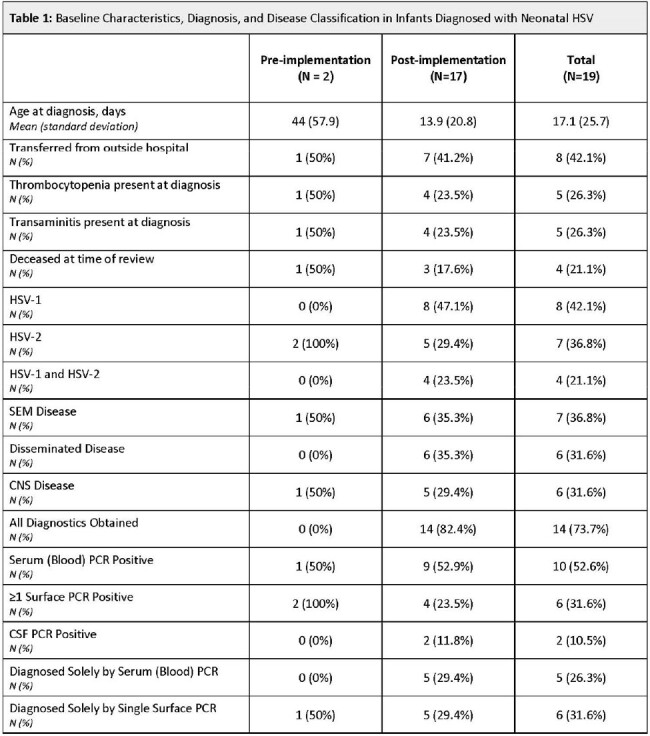
Baseline Characteristics, Diagnosis, and Disease Classification in Infants with Positive Diagnostics

**Methods:**

All infants ≤2 months of age admitted to our institution in the pre-implementation (8/2021-1/2022) or post-implementation (8/2022-5/2023) periods who had at least one HSV PCR obtained were included. Patient demographics and results of HSV testing were collected retrospectively. A complete evaluation was defined as the performance of HSV PCRs from blood, cerebrospinal fluid (CSF), and all surface sites (mouth, both eyes, nasopharynx, and rectum). The diagnosis of neonatal HSV was made by positive diagnostic(s) in conjunction with clinical presentation.

**Results:**

A total of 335 infants (pre-transition=131, post-transition=204) were included. Demographics were similar overall between time periods. Diagnosis of neonatal HSV was made in 2 (1.5%) infants evaluated in the pre-transition and 17 (8.3%) infants evaluated in the post-transition period. Following transition to in-house performance of serum HSV PCR testing, infants were more likely to have a complete evaluation performed (82% vs. 0%; *p*=0.012). Both patients in the pre-cohort had isolated HSV-2 disease while 4 (23.5%) patients in the post-cohort had co-infection with HSV-1 and HSV-2 (**Table 1**). Overall, 5 (29%) patients in in the post-transition period had only a serum HSV PCR positive at diagnosis.

**Conclusion:**

Incidence of neonatal HSV within our institution was higher than previous national estimates and incidence increased following transition to in-house serum testing, possibly due in part to improved rates of complete evaluation. The nonspecific clinical presentation and frequency for which only a single PCR was positive reinforces the importance of collecting all surface, blood, and CSF PCRs in neonates undergoing a sepsis evaluation.

**Disclosures:**

**All Authors**: No reported disclosures

